# Adverse Effects of Perfluorooctane Sulfonate on the Liver and Relevant Mechanisms

**DOI:** 10.3390/toxics10050265

**Published:** 2022-05-19

**Authors:** Pingwei Wang, Dongge Liu, Shuqi Yan, Jiajing Cui, Yujun Liang, Shuping Ren

**Affiliations:** Department of Occupational and Environmental Health, School of Public Health, Jilin University, Changchun 130021, China; wangpw20@mails.jlu.edu.cn (P.W.); liudg21@mails.jlu.edu.cn (D.L.); yansq21@mails.jlu.edu.cn (S.Y.); cuijl20@mails.jlu.edu.cn (J.C.); liangyj20@mails.jlu.edu.cn (Y.L.)

**Keywords:** perfluorooctane sulfonate, PFOS, liver, PFAS, hepatic function, liver injury

## Abstract

Perfluorooctane sulfonate (PFOS) is a persistent, widely present organic pollutant. PFOS can enter the human body through drinking water, ingestion of food, contact with utensils containing PFOS, and occupational exposure to PFOS, and can have adverse effects on human health. Increasing research shows that the liver is the major target of PFOS, and that PFOS can damage liver tissue and disrupt its function; however, the exact mechanisms remain unclear. In this study, we reviewed the adverse effects of PFOS on liver tissue and cells, as well as on liver function, to provide a reference for subsequent studies related to the toxicity of PFOS and liver injury caused by PFOS.

## 1. Introduction

Per- and polyfluoroalkyl substances (PFAS) are organic anthropogenic compounds applied in many aspects due to their characteristics of oil and water repellence, and stability. The pollution of PFAS in the environment arises from its wide use. PFAS are receiving increasing attention due to their properties of stability, bioaccumulation, and long half-life. Recent research shows that perfluorooctane sulfonate (PFOS), one of the major PFAS, is a persistent organic pollutant that is toxic, bioaccumulative, and resistant to degradation [1]. PFOS can be present in the environment persistently and enter and accumulate in human serum, breast milk, liver, and kidney through ingestion and contact with PFOS-containing products and occupational exposure [2]. Liver is the largest digestive organ of human body and also a large metabolic organ of the body. Most toxic substances are metabolized in the liver, and will be excreted from the body through bile or urine. It is reported that PFOS is able to accumulate in liver [3]. However, it is still unknown whether PFOS can be metabolized in the liver and if damage to the liver is caused by PFOS accumulation. Therefore, it is of great significance to summarize the adverse effects of PFOS on liver and their mechanisms due to the ubiquitous nature of PFOS and the vital role of liver in metabolizing PFOS. We searched the studies on PFOS with cells, animals, and population surveys to summarize the current research on the effects of PFOS on liver.

## 2. Profile of PFOS

Perfluorooctane sulfonate (PFOS) is a C8 fluorocarbon compound of per- and polyfluoroalkyl substances (PFAS). PFOS is extremely persistent in the environment and there are no indications that the perfluorinated part of PFOS or PFOA could be decomposed in the environment, which makes PFOS or PFOA ubiquitous in organisms or non-organisms, even in remote areas such as the Qinghai-Tibet Plateau and the polar regions [4]. Since 1950, PFOS has been produced in large quantities around the world, posing a huge threat to the environment and human health [5]. Data for 1998–2015 show that emissions of PFOS are estimated at 1228–4930 tons and data for 1970–2012 show that emissions of PFOS precursors are estimated at 45,000 tons [6,7]. It is reported that PFOS and its precursor showed an increasing trend in the environment with time between 2009–2010 and 2018–2019 [8]. Surveys in densely populated areas of the United States and Europe found that PFOS was detected in water systems at high concentrations ranging between 97 and 1371 ng/L [9]. Moreover, the highest PFOS concentrations observed in some areas of the United States were even as high as 8,970,000 ng/L [10]. In addition, PFOS was detected in 47 fatty fish and 45 shellfish samples collected from six coastal provinces in China [11]. Serum data from 1303 participants recruited from the community of Guangzhou, China, from November 2018 to August 2019 showed a median PFOS of 14.85 ng/mL [12]. PFOS was found in the serum of more than 98% of Americans [13]. A cross-sectional study on lipids and PFOS showed that the levels of serum lipids were closely related to the exposure level of PFOS. The mean levels of serum PFOS in 2005–2006 were 22 ng/mL (median, 20 ng/mL) [14]. Plasma data collected from children in the Boston, MA, USA, area from 2007 to 2010 (median age 7.7 years) showed a median PFOS concentration of 6.2 ng/mL [15]. Data from 2015 to 2016 in the United States showed that the geometric mean of PFOS concentration in serum was 4.72 ng/mL, and the 95% CI was 4.40–5.07 ng/mL [16]. This figure is still close to or even exceeds the PFOS HBM-I value of 5 ng PFOS/mL blood plasma established by the German Human Biomonitoring Committee [17]. For human epidemiological studies and experimental animal studies, exposure to PFOS has been shown to have a variety of health effects on the body, such as hepatotoxicity, infertility, altered thyroid function, decreased immunity, neurotoxicity, developmental toxicity, reproductive toxicity, nephrotoxicity, cardiotoxicity, insulin resistance, and carcinogenic effects [18,19,20,21,22,23,24,25]. Data published by the European Food Safety Agency (EFSA) in 2012 showed that the estimated daily dietary intake of PFOS for adults was 5.2–10 ng/kg body weight (bw) [26]. In 2018, EFSA established a tolerable weekly intake (TWI) of 13 ng/kg bw per week for PFOS based on human epidemiological studies [27]. In 2006, PFOS was considered a priority substance under the EU Water Framework Directive (WFD), which restricts the production and use of PFOS throughout the European Union [28]. As a globally present environmental pollutant, PFOS was recognized as a persistent organic pollutant (POP) in 2009 and was listed in the Stockholm Convention to call for global production restrictions [29]. The Stockholm Convention has been ratified by 179 countries and measures have been introduced to restrict the production and use of PFOS [30,31], and PFOS has been phased out or banned in Canada [32], the United States [33], and the European Union [34]. However, PFOS and its related products continue to be produced and used in some developing countries due to the lack of alternative products or technologies with better cost effectiveness in certain application scenarios [35,36].

Currently, PFOS has been restricted from entering the environment and is even banned from manufacture and usage; however, PFOS is almost everywhere and persistent, which means that human beings will be exposed to PFOS for a long time. The arithmetic and geometric mean half-lives of serum elimination were 5.4 years and 4.8 years for PFOS, respectively [37,38]. Although PFOS is detected at low concentrations in the environment and organisms, it cannot be ignored that PFOS will have overt and adverse effects on human health and organisms due to its persistence and bioaccumulation worldwide [39].

## 3. Relationship between PFOS and the Liver

**Figure 1:**
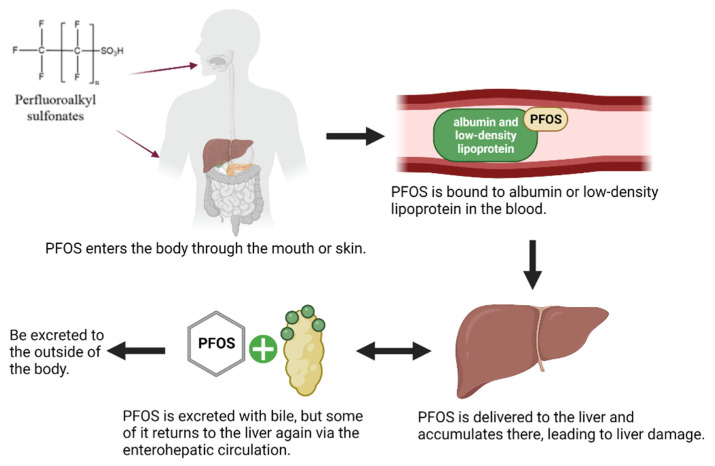
Outline diagram of PFOS entry into the body and excretion from the body.

In the past ten years, the adverse effects of PFOS have emerged and the health risk of PFOS is receiving more attention from researchers [40]. The liver is the most common toxic target and is sensitive to the damage caused by foreign substances [41,42,43,44]. PFOS mainly accumulates in the organs which contain high amounts of proteins such as blood and liver, which results in the higher concentration of PFOS in the liver [45]. PFOS from other organs and blood will be transported to the liver through circulation due to its metabolic characteristics, which leads to the increased accumulation of PFOS in liver and increases the toxic risk to liver. In the human body, PFOS is mainly distributed in blood and extracellular fluid after entering the body, binding to albumin and low-density lipoprotein in blood, and circulates to all organs of the body with blood circulation [46,47]. PFOS is preferentially distributed to and accumulates in the liver during circulating distribution in the body, but it is indicated that PFOS does not seem to be metabolized in the liver or in other parts of the body, nor does it undergo any chemical reactions [48,49]. However, PFOS can be excreted through biliary excretion, and this may be the main route of PFOS excretion in humans [50]. Moreover, after being carried by bile into the intestine, some of the PFOS can be sent back to the liver through the enterohepatic circulation due to the reabsorption of bile, which may be one of the reasons for the high concentration of PFOS in the liver (Figure 1) [2,51]. Wang et al. reported that PFOS could accumulate in the liver gradually and participate in the mediation of gene expression related to lipid metabolism and inflammatory response [52]. The human serum levels of enzyme, such as acetyl coenzyme A, peroxisomal acyl-CoA oxidase 1, acyl-CoA dehydrogenase, and the mitochondrial membrane carnitine-dependent enzyme, were increased after exposure to PFOS, indicating that the liver was damaged [53]. PFOS can cause hepatic steatosis in humans and reduce postnatal survival and growth in rodents [54]. Studies in mice have also shown that PFOS accumulates most in the liver, followed by the lungs, kidneys, spleen, heart, and brain, causing the most serious damage to the liver [55]. An analysis of wild freshwater fish collected from rivers in the Pearl River Delta region of China also demonstrated that PFOS was the major PFAS in fish liver [56]. Therefore, it is particularly important to study the adverse effects of PFOS on liver.

## 4. Effects of PFOS on the Liver and Related Mechanisms of Toxicity

### 4.1. Effect of PFOS on Hepatic Lipid Metabolism

The liver is the main site of fat metabolism in the body and a hub for fat transport. The liver plays a major role in regulating lipid homeostasis through major lipid metabolic pathways, including de novo lipogenesis, fatty acid (FA) synthesis, triglyceride (TG) synthesis, and storage. In addition, there are some complex lipid anabolisms, such as cholesterol, ceramide, phospholipids, lipoprotein synthesis and secretion, and fatty acid oxidation [57]. In the context of human research, in a study that included 290 children (aged 8–10 years) from Taiwan, China, applying lipidomic approaches to examine lipid patterns in the sera of children exposed to different levels of PFAS, the data showed that PFOS was detectable in serum samples from approximately 90% of surveyed children. Upregulation of phosphatidylcholine-containing lipid sphingomyelins (SMs) levels was associated with increased serum PFOS, perfluorotridecanoic acid (PFTrDA), and perfluorodecanoic acid (PFDA) concentration. SMs are thought to be secondary messenger molecules that directly or indirectly influence intercellular or cell–organelle interactions, including insulin resistance, liver dysfunction, and obesity [58]. A recent study on human hepatocytes found that 25 μM PFOS could induce at least 7 transcripts out of 35 related to lipid metabolism, lipid transport, and antioxidant response pathways. Moreover, PFOS induced the expression of genes related to xenobiotic metabolism, such as CYP2B6 and SULT2A1, but no lipid accumulation was observed in hepatocytes [59].

For animal research, toxicological studies have shown that PFOS can induce liver enlargement by directly activating the proliferation of hepatocyte peroxisomes [60]. Moreover, PFOS can induce hepatic steatosis in mice [61]; the mechanism may be that PFOS can cause changes in lipid regulatory genes in the liver, thereby promoting the development of steatosis [62]. A study showed that PFOS administration increases liver weight and expression of genes involved in fatty acid oxidation and transport in rodents [20]. In particular, 10 and/or 5 mg/kg PFOS significantly increases the gene expression levels of fatty acid translocase (FAT/CD36) and lipoprotein lipase (Lpl), and reduces serum levels of very low density lipoproteins, and reduced rates of mitochondrial β-oxidation are also identified [63]. The quantification of gene expression related to lipid metabolism in the mouse liver showed that PFOS caused decreased expression of apolipoprotein genes (ApoA1, ApoA2, ApoB), apolipoprotein packaging gene MTTP, and lipid metabolism gene CES3, but significantly increased PPARγ mRNA levels (PPARγ mRNA is a known nuclear receptor for adipogenesis) in mice [64], which suggested that PFOS could cause the disturbance of hepatic lipid metabolism in mice. Quantification using multilayer glycoproteomics revealed that 241 dysregulated proteins were involved in lipid and xenobiotic metabolism in the liver of PFOS-exposed mice [65]. A study showed that PFOS administration exacerbated the abnormal accumulation of hepatic lipids in mice on a high-fat diet. Moreover, genomic and proteomic analyses showed that PFOS not only induced lipid production and accumulation in mouse liver, but also activated the production of some hepatic transport proteins (for example, OATPs and NTCP, two transporter proteins that have been shown to be closely associated with the transport clearance of PFOS [66]) and stimulated the metabolic pathways to accelerate the clearance of lipids and PFOS in the body [67]. A similar conclusion was drawn in the study by Marisa Pfohl et al. that PFOS increased the expression of target genes and proteins related to lipid metabolism and oxidative stress in the liver [68]. It is hypothesized that PFOS may increase the risk for hepatic metabolic and inflammatory diseases by inducing dysregulation of lipid metabolism and activating oxidative stress [69]. Paradoxically, lipid accumulation was found in rodent-related experiments after PFOS exposure, but not in human-related experiments, which may be attributed to the disparity between species, but these reasons need to be further explored in the future. Another study in zebrafish showed that chronic PFOS exposure induced hepatic steatosis in zebrafish by interfering with lipid biosynthesis, fatty acid β-oxidation, and VLDL/LDL lipoprotein excretion. Further studies showed that PFOS significantly increased the expression of nuclear receptor (NR) and fatty acid oxidation-related genes, and that disruption of NRs regulation contributed to the development and progression of several liver diseases, including fatty liver and liver fibrosis [70]. These results suggest that PFOS can cause damage to the liver by causing abnormalities in lipid metabolism in the liver.

### 4.2. Direct and Indirect Effects of PFOS on Maternal and Offspring Livers

In the context of human research, it has been shown that PFAS may have genotoxic and cytotoxic potential for human hepatocytes [71]. This conclusion is also supported by a European cohort study in which exposure to PFAS, including PFOS, during fetal development can cause liver injury in children [72]. In a study to detect concentrations of PFAS in fetal and corresponding placental samples and maternal serum samples from elective terminations and intrauterine deaths, it was shown that PFOS was found in all samples and tissues; the sum of all PFASs was highest in livers from mid- and late-pregnancy samples [73], which suggests that PFAS, including PFOS, can cross the placental barrier and deposit into embryonic and fetal tissues, and cause damage to their livers [74]. The results from transcriptome sequencing and bioinformatics analysis show that prenatal PFOS exposure activates fatty acid and lipid synthesis and metabolism, leading to liver damage and interfering with fetal liver development; moreover, several pro-cancer signaling pathways, including Wnt/β-catenin, Rac, and TGF-β, were also activated in fetal liver [75], which suggests that prenatal exposure to PFOS is not only hepatotoxic to the mother, but also leads to long-term liver disease in the offspring. Therefore, it is important to perform PFOS exposure assessments in women during pregnancy.

In terms of animal research, a mice experiment with utero exposure to PFOS has shown that significant hepatomegaly and steatosis occurred in the PFOS group of female rats, and increased triglyceride, total cholesterol, and LDL content and decreased HDL content were found in the liver of postnatal day 1 (PND1) mice; further analysis yielded significant changes in the expression of functional messenger RNAs for PND1 hepatocytochrome P4A14 (CYP4A14), CD36 (hepatic fatty acid uptake), apolipoprotein B100 (APOB), and fibroblast growth factor 21 (FGF21) [76]. In another study on fish chronically exposed to PFOS, it was demonstrated that long-term exposure to PFOS could produce adverse effects on lipid metabolism in both F0 and F1 generations [77]. PFOS damaged the liver structure of juvenile marine medaka by enlarging the nucleus and disrupting the cell structure, and acute exposure to PFOS affected the expression of cirrhosis-related genes at the transcriptional level [44,62]. In addition, studies in Australian finfish pups have shown that PFOS can be transferred from mother to pup through the placenta and during lactation, and can cause damage to the pups’ liver [78]. Moreover, placental transfer is the main source of PFAS in hooded seal pups and is thought to be the main route of excretion of PFAS in adult females [79]. From the above studies, we can see that parental PFOS exposure can be transferred to the liver of the zygotic embryo and fetus, thus causing a disturbance in the lipid homeostasis of the zygotic hepatocytes and leading to the occurrence of liver damage.

### 4.3. Effect of PFOS on Biomarkers of Liver Function

An analysis of the association between perfluorinated alkyl acid (PFAA) exposure, including PFOS, and biomarkers of liver function in adolescents (12–19 years of age) from the 2013–2016 National Health and Nutrition Examination Survey (NHANES) shows that individual serum perfluoroalkyl acids (PFAA) are closely correlated with markers of liver function, including alanine aminotransferase (ALT), aspartate aminotransferase (AST), and gamma-glutamyl transpeptidase (GGT), which can be used as a basis for analyzing the association between PFOS exposure and liver injury [80]. We determine abnormal liver function by levels of one or more biomarkers of liver function that exceed their upper limit of normal (ULN). PFOS was found to be positively correlated with alanine aminotransferase (ALT, a sensitive marker of acute hepatocellular damage) activity, and every 1 ln unit increase in PFOS exposure was associated with a 4.3% (95% CI: 1.2%, 7.4%) increase in ALT levels and a 33.0% (95% CI: 5.0%, 67.0%) increase in the odds of abnormal ALT [81,82,83,84]. In addition, recent data from China showed a positive correlation between serum concentrations of PFOS and ALT and AST levels [12]. However, this contradicts U.S. data from 2007 to 2010, where serum data showed no significant association between PFOS and AST [13]. Canadian data surprisingly showed no correlation between PFOS and either AST or ALT [85]. We speculate that this may be related to differences in region and ethnicity, and this needs to be explored further. In addition, in animal experiments, PFOS administration increased serum concentrations of ALT and AST in mice [86].

In addition, PFOS was negatively correlated with the levels of tumor necrosis factor alpha (TNFα) and interleukin 8 (IL-8), and further studies revealed that PFOS exposure was strongly associated with increased hepatocyte apoptosis biomarkers (e.g., CK18-M30) and decreased serum TNFα [87]. Previous studies from cohorts and experiments suggest that PFOS may cause liver injury and downregulate certain indicators of immune response.

### 4.4. Effect of PFOS on Hepatocyte Proliferation

In the area of human cell research, exposure of a human hepatocyte cell line (HL-7702) to 50 μM PFOS identified 52 differentially expressed proteins, 27 of which were associated with cell proliferation, including hepatoma-derived growth factor (Hdgf) and the proliferation biomarkers nuclear protein Ki67(Ki67) and topoisomerase 2 alpha (Top2α). Low doses of PFOS (below 200 μM) enhanced the expression of Hdgf and Ki67 and Top2α [88]. Another study showed that PFOS could promote cell differentiation and cell proliferation by reducing positive targets such as cholesterol 7 alpha-hydroxylase (CYP7A1) in differentiation genes, and increasing negative targets such as Cyclin D1 (CCND1) in mitogenic genes through the study of hepatocyte nuclear factor 4-α (HNF4α) target gene expression [89].

In animal studies, PFOS exposure upregulated the expression of PCNA, c-Jun, c-MYC, and Cyclin D1 in rat Kupffer cells and co-cultured rat primary hepatocytes. The mechanism may be that PFOS induces Kupffer cell activation and leads to hepatocyte proliferation via the NF-κB/TNF-α/IL-6-dependent pathway [86].

### 4.5. Effect of PFOS on Liver Tumors

Cancer is the second leading cause of death worldwide, with primary liver cancer being the sixth most commonly diagnosed cancer and the third leading cause of cancer death worldwide in 2020 [90]. The liver is the primary target organ for PFOS exposure [91]. Epidemiological surveys reveal that PFOS is associated with the risk of developing cancers [92]. In a study on the relationship between PFAS concentrations in human serum and liver cancer from 2019 to 2021, the highest serum concentrations of PFOS were found in different PFAS with a 100% detection rate, and the concentration of PFOS in human serum was found to be significantly associated with the incidence of liver cancer and the levels of alpha fetoprotein (AFP), indicating that exposure to PFOS can increase the risk of developing liver cancer [93].

Animal studies display that PFOS exposure increases the risk of liver tumorigenesis [94]. A study on transgenic zebrafish showed that PFOS caused liver enlargement in model fish and increased the likelihood of hepatocellular carcinoma (HCC) in zebrafish [95]. The results also showed that PFOS led to reduced synthesis and accelerated degradation of the active form of vitamin D, osteotriol, resulting in a decrease in intracellular osteotriol content. In addition, the population study found that the level of vitamin D in human serum was negatively correlated with the concentration of PFOS [96].

### 4.6. Effect of PFOS on Hepatic Immune Function

PFOS could suppress the expression levels of almost all immune-related genes (such as interleukins and interleukin receptors, cellular chemokine family and NF-κB family, etc.), suggesting that these genes may be involved in the negative regulation of the body’s immune response following PFOS exposure, which results in a decrease in immunity [97]. Exposure of rodents to PFOS results in marked hepatomegaly, which is associated with significant alterations in liver histophysiology and immune status [98]. Moreover, a study has shown that PFOS exacerbates acute liver injury caused by concanavalin A (Con A), and the injury is associated with reduced levels of the liver pro-inflammatory cytokines tumor necrosis factor alpha (TNF-α) and interferon gamma (IFN-γ). In addition, exposure to PFOS can enhance hepatic DNA fragmentation, particularly after short-term exposure to low doses of PFOS, which suggests that exposure to PFOS may enhance the susceptibility of hepatic parenchymal cells to other injuries caused by activation of the hepatic immune system, thereby exacerbating liver injury during acute inflammation [99]. It was shown that PFOS at 1 μM significantly reduced the immune-related gene il1β in zebrafish liver and correspondingly reduced the protein level of IL-1β, with an increased binding potential to NF-κB [100]. In a study of striped bass, elevated PFOS concentrations were associated with increased lysozyme and AST activity, suggesting that PFOS exposure affects hepatic immune function [101]. In a study on male zebrafish, it was shown that the activities of immune-related enzymes such as acid phosphatase (ACP), alkaline phosphatase (AKP), and myeloperoxidase (MPO) were inhibited and the activity of lysozyme (LSZ) was increased after PFOS exposure. The mRNA levels of immune-related factors IL-1β, IL-6, IL-15, TNF-α, TGF-β, IRF-1, IRF-3, IRF-7, RAG-1, and RAG-2 were significantly increased in a concentration- and time-dependent manner with increasing PFOS exposure, suggesting that PFOS can interfere with normal immune function in zebrafish [102,103,104].

### 4.7. Effect of PFOS on Cholesterol and Bile Acid Metabolism

**Figure 2:**
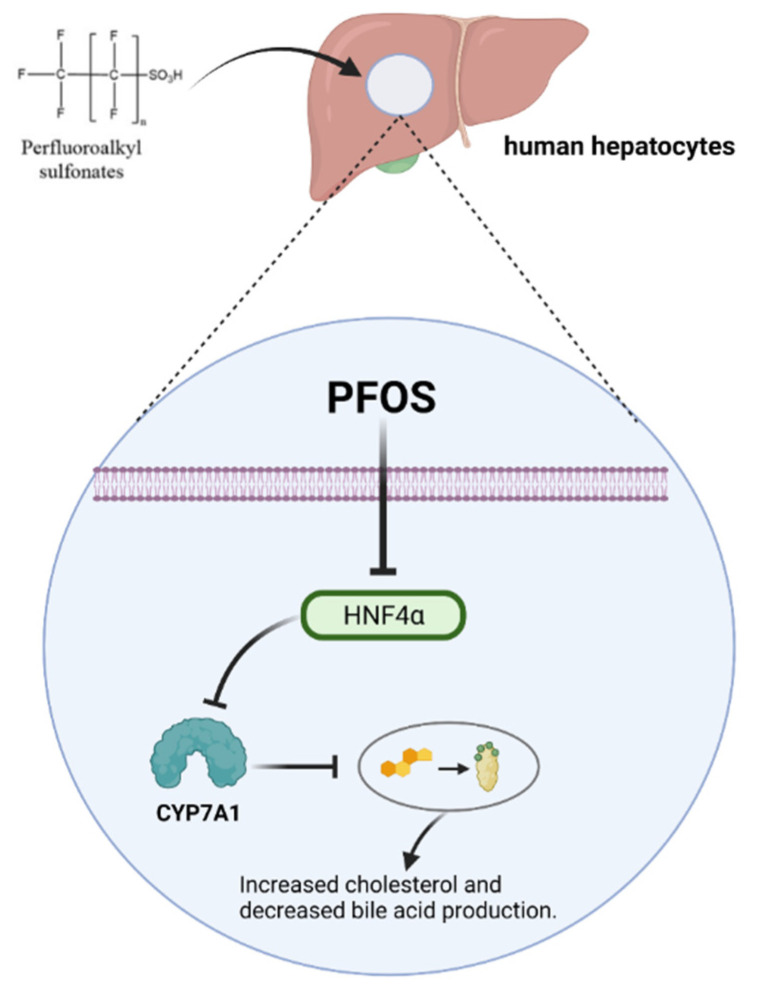
PFOS affects the metabolism of cholesterol and bile acid via CYP7A11.

The European Food Safety Authority (EFSA) has identified exposure to PFOS as an important factor contributing to elevated total serum cholesterol levels in humans [27]. In the context of human research, epidemiological studies have also demonstrated that PFOS alters the transcript levels of genes associated with cholesterol mobilization [105]. PFOS has been shown to reduce serum cholesterol levels in rodents [106]. In a study based on hepatic HepaRG cells, it was found that the expression of numerous genes involved in the synthesis, metabolism, and transport of cholesterol and bile acids in cells was significantly affected by PFOS at concentrations above 10 µM, and there was a significant decrease in the synthesis of bile acids. In particular, PFOS leads to significant downregulation of the gene and protein levels of CYP7A1, the key enzyme that catalyzes the first and rate-limiting step in the synthesis of bile acids from cholesterol [107]. PFOS causes a decrease in HNF4α protein expression in human hepatocytes, a transcription factor that regulates CYP7A1, and a decrease in CYP7A1 leads to a decrease in bile acid production and an increase in cholesterol (Figure 2) [89,108].

In terms of animal research, another study in estrogen receptor β (ERβ) knockout mice exposed to PFOS revealed that PFOS significantly triggered hepatocyte edema and vacuolization, reduced bile acid and cholesterol levels in liver tissue, and affected the abundance and composition of the intestinal microbiota [60].

## 5. Signaling Pathways Associated with Hepatotoxic Damage Caused by PFOS

### 5.1. Role of Inflammation-Associated Signaling Pathways in PFOS-Induced Hepatotoxic Damage

Using liver lipidomic analysis in mice, it was found that phosphatidylcholine (PC) synthesis via the phosphatidylethanolamine N-methyltransferase (PEMT) pathway may be a self-protective mechanism against PFOS-induced inflammation in animals [55]. PC has a variety of beneficial effects, including anti-inflammatory, antioxidant, and anti-fibrotic activities. Increased PC levels can be used to combat inflammation caused by PFOS. PC is upregulated in the PFOS-exposed state, and the upregulated PC may mainly originate from the PEMT pathway. A study showed that PC was increased in chicken embryos exposed to PFOS and was mainly derived from the PEMT synthesis pathway [109].

**Figure 3:**
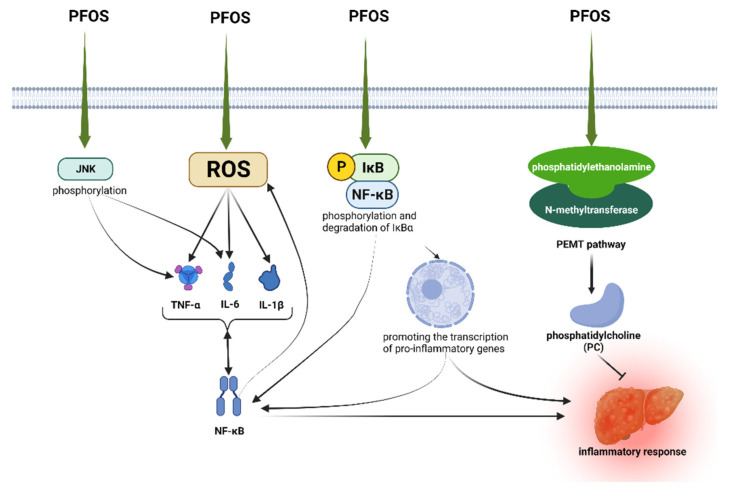
Summary diagram of inflammation-related pathways mediated by PFOS.

NF-κB is thought to be an important regulator of pro-inflammatory gene expression and can be activated by various inflammatory stimuli and environmental stressors, including IL-1β, and be transferred from the cytoplasm to the nucleus, thereby promoting the transcription of pro-inflammatory genes [110]. It has been shown that PFOS induces phosphorylation and degradation of IκBα, which will facilitate the translocate NF-κB from the cytoplasm to the nucleus and activate the NF-κB signaling pathway in C6 glioma cell lines. Thus, the NF-κB signaling pathway can be activated after PFOS exposure [104]. Another study found that PFOS exposure increased ROS levels; increased the mRNA expression of IL-1β, IL-6, and TNF-α; and enhanced NF-κB activity in the liver [102]. In addition, PFOS can increase both TNF-α and IL-6 expression by activating the NF-κB signaling pathway, thus affecting the regulation of the immune system [103]. PFOS induces IκB and JNK phosphorylation and activates NF-κB in the liver and induces TNF-α and IL-6 release by regulating JNK and NF-κB signaling pathways in Kupffer cells [86]. Moreover, significant changes in PFOS-induced NF-κB and IκBα protein expression were found to be associated with elevated TNF-α levels [111] (Figure 3).

### 5.2. Role of Oxidative Stress-Related Signaling Pathways in PFOS-Induced Hepatotoxicity

**Figure 4:**
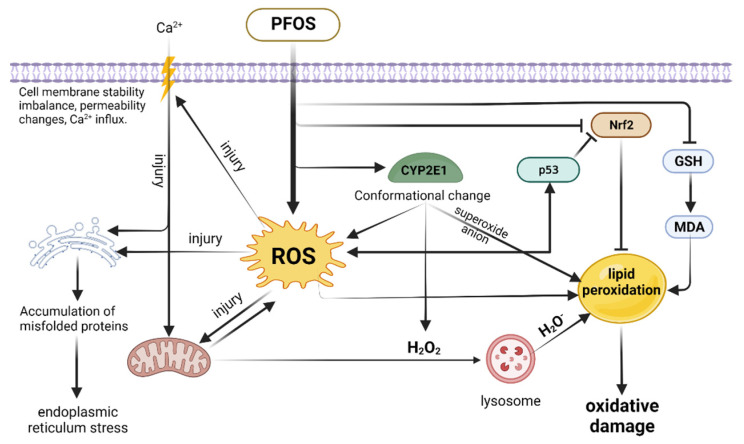
Outline diagram of ROS-related pathways mediated by PFOS.

The liver is particularly sensitive to ROS-induced oxidative stress [112]. PFOS exposure is able to increase ROS production in the liver and disrupt intracellular antioxidant defense systems [113]. ROS, an important pathogenic factor in the pathogenesis of many liver diseases, is usually a by-product of hepatocyte metabolism and detoxification. Continuous and excessive ROS production is highly toxic to cells and leads to oxidative damage to biomolecules in hepatocytes [112]. PFOS can directly damage hepatocyte mitochondria by disrupting the electron transport chain and releasing the toxic ROS. Then, hydrogen peroxide (H_2_O_2_), resulting from the damaged mitochondria and the conformational change in CYP2E1 caused by PFOS, diffuses into the lysosome and leads to the production of highly reactive hydroxyl radicals (H_2_O^−^) [114,115]. CYP2E1 produces superoxide anions during its catalytic cycle, and these ROS can subsequently destroy unsaturated fatty acids, leading to lipid peroxidation and the production of highly reactive aldehydes, such as malondialdehyde [116,117,118]. PFOS exposure promotes the accumulation of H_2_O_2_ and increased MDA content, leading to a decrease in SOD and GSH levels. Furthermore, PFOS can cause oxidative damage in the liver by inducing ROS formation and depletion of anti-oxidative defenses [119]. Excess ROS can attack the cell membrane of hepatocytes, leading to imbalance in cell membrane stability and altered permeability. The eventual disintegration will be triggered by lipid peroxidation caused by ROS [120]. ROS can cause an increase in Ca^2+^ entering the hepatocytes, which in turn leads to an increase in Ca^2+^ in the organelles, ultimately leading to organelle and overall cellular damage [121]. ROS-induced oxidative stress can induce the occurrence of mitochondrial permeability transition (MPT) in hepatocytes, which also leads to changes in mitochondrial membrane potential [122,123]. Moreover, mitochondrial ROS can escape from the MPT process, which in turn increases the intensity of oxidative stress [124]. In addition, the imbalance of Ca^2+^ homeostasis caused by ROS interferes with the function of the endoplasmic reticulum, leading to the accumulation of misfolded proteins and the enlargement of the endoplasmic reticulum lumen, and ultimately to endoplasmic reticulum stress [125,126]. Nuclear factor erythroid 2-related factor 2 (Nrf2) is a key transcriptional activator that provides essential protection against cellular oxidative damage by regulating the expression of antioxidant and detoxification enzyme genes, and is considered a key node in the prevention and treatment of liver disease [127]. PFOS can inhibit the activation and expression of target genes of Nrf2 [128]. PFOS can also inhibit Nrf2 expression in rat liver [111]. The tumor suppressor gene p53 is an important transcription factor that regulates the level of cellular ROS. High levels of ROS or prolonged external stimuli upregulate p53 and trigger a pro-oxidative response to further increase ROS expression [129]. Upregulated p53 expression decreases Nrf2 protein levels and subsequently inhibits Nrf2-dependent antioxidation and triggers p53-regulated apoptosis [130]. It was found that PFOS could inhibit the expression of Nrf2 through ROS-induced p53 dysregulation and thus enhance the level of oxidative stress in cells [119]. Exposure of hepatocytes to PFOS induces rapid depletion of glutathione (GSH), an important marker of cellular oxidative stress, and hepatotoxicity of PFOS can be inhibited by antioxidants and ROS scavengers [114] (Figure 4).

### 5.3. Role of PPAR-Related Signaling Pathway in Hepatotoxic Damage Caused by PFOS

Peroxisome proliferator-activated receptors (PPARs) have four main subtypes: PPARα, β, δ, and γ. PPARs are nuclear receptors involved in the regulation of genes related to energy homeostasis, lipid metabolism, and inflammation [131,132]. Studies have shown that PFOS can activate PPAR in the liver, thereby disrupting cholesterol homeostasis and fatty acid ω-oxidation [133]. PPARα-null mice treated with various PFAS exhibit steatosis characterized by the accumulation of lipids in hepatocytes, leading to liver disease [134]. A dose-dependent increase in PPARα-regulated gene expression upon PFOS exposure has been observed in rodents [135]. PFOS increased the expression of PPARα target genes Acox1 and CYP4A10 in mouse liver, thereby activating PPARα and causing pathological changes in the liver tissue [136]. Recently, in a study using a primary human hepatocyte spheroid model, PPARα activity was found to be increased by PFAS exposure in human liver spheroids, particularly by PFOS exposure [137]. Previous studies have shown that PFOS can differentially activate PPARα in mice and humans [138,139]. Prolonged activation of PPARα also increases signaling of hepatocyte proliferation and causes hepatocellular carcinoma in rodents, but PPARα-induced hepatocellular carcinoma appears to occur only in rodents, and there are species differences between rodents and humans in this aspect [140,141,142]. Currently, most researchers agree that liver cancer caused by PPARα does not seem to occur in human cells [143]. It is hypothesized that human PPARα may have lower sensitivity to PPARα agonists compared to rodent [136]. Therefore, more studies are needed to explore the relationship between PFOS, human liver, and PPARα in the future. A study of genes related to lipid metabolism in developing chicken embryos exposed to PFOS revealed that transcription of genes involved in fatty acid oxidation and PPAR-mediated were extensively suppressed, and the effect was more pronounced at lower doses of PFOS exposure [144].

### 5.4. Role of Autophagy-Related Signaling Pathways in PFOS-Induced Hepatotoxic Damage

Autophagy, as a self-regulated catabolic pathway, plays a key role in energy homeostasis and cytoplasmic quality control. For example, autophagy regulating hepatocyte function and autophagy dysregulation is associated with many liver diseases [145]. Previous studies have shown that PFOS can cause cells to undergo dysregulation of oxidative homeostatic change, leading to abnormal autophagy [146]. PFOS can induce abnormal expression of autophagic markers LC3-II and P62 and increase autophagic flux [147]. For the liver, PFOS induced LC3-II and LC3-II/I expression in L-02 cells in a concentration-dependent manner, stimulated autophagic flux, and decreased p62 expression [148]. It was also found that PFOS altered the post-translational modification of α-tubulin associated with autophagy in L-02 cells, but no changes in α-tubulin were found in the livers of PFOS-treated mice [149]. However, the mechanism is unclear and needs to be further explored. Another study showed that PFOS increased the number of autophagosomes in HepG2 cells and elevated LC3-II expression levels, induced lysosomal membrane permeabilization (LMP), and subsequently blocked autophagic flux, leading to excessive accumulation of autophagosomes and ultimately cellular damage [150]. These studies suggest that PFOS can trigger the disruption of mechanisms associated with autophagy in the liver, ultimately leading to liver damage.

### 5.5. Role of Apoptosis-Related Signaling Pathways in Hepatotoxic Damage Caused by PFOS

Apoptosis is a physiological form of programmed cell death that plays a critical role in embryogenesis, development, and normal tissue homeostasis [151]. PFOS has been reported to disrupt the homeostasis of the antioxidant system in hepatocellular carcinoma HepG2 cells; to promote the production of ROS, which in turn affects mitochondrial function; and to influence the gene expression of apoptosis regulators, leading to the initiation of the apoptotic program [152]. ROS are mainly derived from mitochondria in cells [153] in response to environmental stimuli. Excess ROS inhibit mitochondrial membrane potential (MMP) and trigger downstream apoptotic effects [154]. PFOS increases ROS levels in hepatocytes in a concentration-dependent manner, enhances Bax and cleaved-caspase-3 expression, inhibits MMP, and reduces Bcl-2 expression and the Bcl-2/Bax ratio to cause apoptosis [148].

**Figure 5:**
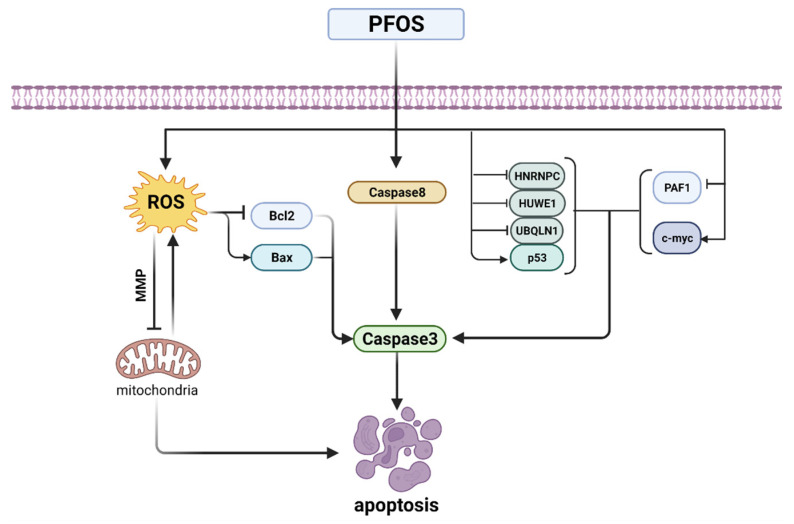
Summary diagram of PFOS-induced apoptotic pathways.

Transcriptional activation of the tumor suppressor gene p53 and the proto-oncogene c-myc have been well documented to be associated with the apoptotic process [155]. PFOS upregulates the gene expression levels of p53 and c-myc in L-02 cells. The suppression of p53-interacting proteins HNRNPC, HUWE1, and UBQLN1 and the overexpression of c-myc-interacting protein PAF1 may contribute to PFOS-induced apoptosis in L-02 cells [156]. The p53 protein plays a key role in cell cycle arrest and apoptosis [157]. PFOS significantly increases p53 mRNA levels and protein expression, which in turn initiates caspase3, the executioner in the caspase family of downstream apoptosis mechanisms, leading to apoptosis [158]. In addition, another study also confirmed that PFOS increased the expression of caspase3 and caspase8 in rat hepatocytes, leading to disrupted gene expression, oxidative damage, and apoptosis in hepatocytes [159] (Figure 5).

## 6. Discussion

In recent years, more and more attention has been paid to the widespread presence of PFOS in the environment and its pollution of the environment, and the health risks posed by PFOS have also attracted increasing attention. As previously stated, increasing evidence suggests that the liver is the primary target organ for the toxic effects caused by PFOS [91]. In this paper, we summarized literature that explores the relationship between PFOS exposure and pathological changes and functional effects on the liver. Although we have described many possible mechanisms for the toxic effects of PFOS on liver tissues or cells, the mechanism of PFOS damage to the liver is not fully understood and needs to be further investigated.

PFOS can disrupt the balance of liver lipid metabolism, resulting in a series of morphological and functional pathological changes in the liver, such as liver enlargement and liver steatosis, and leading to liver diseases such as fatty liver and liver fibrosis, and in severe cases, even liver tumors [160]. In addition, PFOS can cross the placental barrier and have direct and indirect effects on the liver of both parents and offspring. The effects on liver function, hepatocyte proliferation, liver tumors, liver immunity, and cholesterol and bile acid metabolism cannot be ignored. There is substantial evidence that impaired structure and function of liver tissues or cells can affect the function of other tissues and organs; for example, emerging changes in renal function in patients with acute or chronic liver disease often lead to acute kidney injury [161] or chronic kidney diseases [162]. Severe abnormalities in liver function can even damage the nervous system and cause central nervous system dysfunction, leading to the development of hepatic encephalopathy [163]. Abnormal liver function can also affect cardiac function, and patients with cirrhosis can develop increased left ventricular weight and left eccentric hypertrophy, leading to the development of cirrhotic cardiomyopathy [164]. Liver damage can also affect the function of some other organs and tissues; therefore, the mechanism of PFOS damage to the liver is relatively complex, and there are limitations in the independent exploration of the mechanism of PFOS damage to the liver. It is also important to note that the mechanisms of cellular transformation and the extent of damage observed in humans and other animals, especially in rodents, are different due to species differences after PFOS exposure, and therefore, we must be careful when applying the results obtained from animal experiments to human studies, especially in terms of genotoxicity, bioaccumulation, and cytotoxicity [165].

The signaling pathways involved in the toxic damage of PFOS to liver are mainly the inflammation-related signaling pathway, oxidative stress signaling pathway, PPAR-related signaling pathway, autophagy-related pathway, and apoptosis-related signaling pathway, among which ROS homeostasis and NF-κB activation are the key nodes. Several signaling pathways play roles in liver function, but the exact mechanisms of toxicity underlying liver damage after PFOS exposure remain unclear. For example, the Wnt/β-catenin pathway regulates liver development, differentiation, and compartmentalization, and regulates basic liver physiological functions, but excessive activation of the Wnt/β-catenin pathway can lead to the development and progression of hepatocellular carcinoma (HCC) [166]. The PI3K/AKT pathway can lead to insulin resistance and abnormal hepatic gluconeogenesis in the liver [167]. These findings provide new directions for further research in the future. Different signaling pathways act individually or in association with other signaling pathways, forming a complex regulatory network of signaling pathways that regulate the expression of every function in our organisms. PFOS can disrupt the blood–testis barrier by downregulating connexins through the p38 MAPK/ATF2/MMP9 signaling pathway [168], and also mediates nitric oxide production in HAPI microglia via the ROS-mediated ERK/JNK MAPK signaling pathway [169]. These results suggest that PFOS does not affect a single signaling pathway and function in body tissues alone, and that these signaling pathways are not independent of each other, but are interconnected. Therefore, the effects of PFOS on the liver through various signaling pathways are complex.

Due to the stable nature of PFOS and the fact that they are not easily decomposed, they are widely present in environmental media and pose a great threat to the life and health of living organisms. Moreover, PFOS may interact with other environmental pollutants and synergistically cause damage to organisms. Differences in species, age, exposure time, and exposure dose pose many difficulties in studies of PFOS toxicity to the liver. Therefore, focusing on the effects of PFOS on the liver function of the organism in different species and at different time points, and the interactions among toxicants, contribute to future exploration of the mechanisms of PFOS toxicity and provide a theoretical reference for related studies on PFOS-induced hepatotoxicity.
